# The incidence of orthostatic intolerance after bariatric surgery

**DOI:** 10.1002/osp4.383

**Published:** 2019-12-06

**Authors:** James B. Zhang, Robyn A. Tamboli, Vance L. Albaugh, David B. Williams, Donna M. Kilkelly, Carlos G. Grijalva, Cyndya A. Shibao

**Affiliations:** ^1^ Department of Surgery Vanderbilt University Medical Center Nashville Tennessee; ^2^ Department of Health Policy Vanderbilt University Medical Center Nashville Tennessee; ^3^ Mid‐South Geriatric Research Education and Clinical Center VA Tennessee Valley Health Care System Nashville Tennessee; ^4^ Department of Medicine Vanderbilt University Medical Center Nashville Tennessee

**Keywords:** bariatric surgery, incidence, orthostatic hypotension

## Abstract

**Aims:**

Every year, over 200 000 individuals undergo bariatric surgery for the treatment of extreme obesity in the United States. Several retrospective studies describe the occurrence of orthostatic intolerance (OI) syndrome after bariatric surgery. However, the incidence of this syndrome remains unknown.

**Materials and methods:**

We used a prospective, de‐identified registry of 4547 patients who have undergone bariatric surgery at Vanderbilt to identify cases of new‐onset OI. Structured chart reviews were conducted for all subjects who reported new‐onset OI post surgery. Cases of OI were confirmed using an operational case definition developed by the Vanderbilt Autonomic Dysfunction Center, and autonomic function tests results were examined for evidence of impaired autonomic function. The cumulative incidence of post‐bariatric surgery OI syndrome was estimated using a life table.

**Results:**

Seven hundred forty‐one of 4547 (16.3%) patients included in our cohort reported new OI symptoms after surgery. After the chart review, we confirmed the presence of post–bariatric surgery OI syndrome in 85 patients, 14 with severe OI requiring pressor agents. At 5 years post surgery, follow‐up is reduced to 15%; the unadjusted 5‐year prevalence of OI was 1.9%. The cumulative incidence of OI syndrome adjusted for loss of follow‐up was 4.2%. Most OI cases developed during weight‐stable months (±5 kg). At the time of identification, 13% of OI cases showed evidence of impaired sympathetic vasoconstrictor activity.

**Conclusion:**

OI is frequent in the bariatric population, affecting 4.2% of patients within the first 5 years postoperatively. In 13% of post–bariatric surgery OI patients, there was evidence of impaired sympathetic vasoconstriction activity.

## 1 INTRODUCTION

Obesity afflicts over 117 million American adults, and the cost of its care amounts to over $340 billion annually.^1^ Bariatric surgery is the most effective and only treatment modality for obesity that results in sustained weight loss and improvement of glucose control in type 2 diabetes mellitus (T2DM) and cardiovascular risk factors.^2^, ^3^

It is estimated that over 200 000 patients in the United States undergo bariatric surgery every year,^4^ and therefore, it becomes increasingly important to understand the long‐term consequences of this operation. In this context, small case series studies^5^, ^6^, ^7^ have documented the presence of new‐onset orthostatic intolerance (OI) in postoperative bariatric surgery patients.

OI is a rare and chronic disabling condition characterized by dizziness, lightheadedness, and syncope on standing that improve after sitting or lying down.^8^ This disorder significantly reduces the quality of life,^9^ and in severe cases, it requires the use of pressor agents to control the presyncopal symptoms.

The diagnosis of OI is challenging and requires the identification of chronic pre‐syncopal symptoms on standing not related to the use of medications or conditions associated with blood volume depletion. Hence, it is not surprising that previous studies that examined the long‐term consequences of bariatric surgery did not report OI as significant adverse events.^10^ The burden of OI in patients that underwent bariatric surgery is unknown.

The goal of this study was to estimate the incidence of OI and its temporal relationship with weight changes among patients who underwent bariatric surgery. For this purpose, the authors assembled a large retrospective database from a high‐volume bariatric surgery centre in the Southeast United States. OI cases were ascertained using a protocol developed by the Vanderbilt Autonomic Dysfunction Center (VADC), a national referral centre for patients with OI, and the cases were adjudicated after an exhaustive chart review.

## 2 MATERIALS AND METHODS

### 2.1 Data sources

The current study was conducted using the Vanderbilt Metabolic and Bariatric Surgery Quality, Efficacy, and Safety Database (VMBSD), which is a prospective de‐identified registry that includes records of all patients who have undergone bariatric surgery at Vanderbilt University Medical Center (VUMC). The data not available in the VMBSD were extracted through medical chart review using the Vanderbilt Synthetic Derivative (SD), a de‐identified database containing additional clinical and procedural information derived from Vanderbilt's electronic medical records system.^11^ The SD database search interface allows users to find basic clinical and demographic data, such as ICD 9 and 10 codes, current procedural terminology (CPT) codes, medications, laboratory values, age, gender, and vitals in the records. Records in both databases can be linked using internal identifiers. The Vanderbilt Institutional Review Board approved the study.

### 2.2 Patients with bariatric surgery

Within VMBSD, data were queried from 1997 to 2017 to identify patients who underwent bariatric surgery on the basis of CPT codes. Patients who had undergone Roux‐en‐Y gastric bypass (RYGB) were identified through CPT codes 43644, 43645, 43846, or 43847, and patients who had undergone vertical sleeve gastrectomy (VSG) were identified through CPT code 43775.

### 2.3 Review of medical records

The SD medical records were queried of bariatric surgery patients for symptoms of OI and identified cases in two stages. In the first stage, potential patients with OI were identified on the basis of recorded ICD9 or ICD10 diagnosis codes for dizziness, orthostatic hypotension, tachycardia, bradycardia, or syncope coded after the operation (Appendix S1). In the second stage, the records of these potential patients with OI were reviewed via natural language processing for final determination of OI diagnosis.

### 2.4 Definition of OI

The VADC, a national referral centre for autonomic disorders of blood pressure regulation, developed an adjudication protocol. The protocol was designed to identify OI patients on the basis of chart review following published guidelines.^8^ During the chart review, OI was defined as the presence of symptoms like dizziness, lightheadedness, and syncope upon standing at least 30 days after the operation with a duration of at least 3 months, with haemodynamic changes (orthostatic hypotension, tachycardia, and bradycardia). Participants with pathological conditions reported preoperatively or postoperatively that could explain the occurrence of OI symptoms as previously reported by our group (ie, haemorrhage, dehydration, and anaemia) were excluded.^12^ Similarly, those on new medications or changes in the dose of medications known to cause or worsen OI (diuretics, alpha‐1 blockers, or vasodilators) were excluded.

For participants with confirmed postoperative, new‐onset OI, we defined the time of diagnosis to be the earliest time when all the adjudication conditions above were fulfilled. Patients whose time of diagnosis exceeded 72 months post operation were deemed to have developed OI for reasons unrelated to the bariatric operation and were excluded. Furthermore, patients were classified as having “severe” OI if they were prescribed the vasopressor agents midodrine or fludrocortisone. All patients who were not prescribed these agents were designated as having “moderate” OI.

### 2.5 Assessment of medical records review reliability

Before chart review, authors J.B.Z. and D.K. underwent comprehensive training on the adjudication protocol by C.A.S., a board‐certified autonomic specialist. At the end of the training, 50 charts were independently reviewed by C.A.S, J.B.Z., and D.K. The agreement between independent reviewers had a Kappa statistics of 0.8. The reviewed medical records were designated as “OI,” “No OI,” or “Indeterminate.” Patients initially designated as “Indeterminate” by the main reviewer were examined and ultimately adjudicated as “OI” or “No OI” by C.A.S.

### 2.6 Identification of comorbidity and demographic data

Study patients were characterized using data on gender, race, ethnicity, and age at the time of operation and using ICD 9 and 10 diagnosis codes to identify relevant comorbidities recorded in the medical record before bariatric surgery. Study comorbidities included T2DM, hypertension, end‐stage renal disease (ESRD), sleep apnoea, coronary artery disease (CAD), and hypoglycaemia. Neuropathy was defined using the specific ICD 9 and 10 codes and the use of gabapentin with or without abnormal electromyography (EMG) test. Congestive heart failure was defined using the specific ICD 9 and 10 codes and the use of diuretics. All the ICD 9 and 10 codes are listed in Appendix S2.

### 2.7 Body weight data

Available body weight data from bariatric surgery patients who returned for a follow‐up visit were collected at 0, 0.3, 1, 3, 6, 9, 12, 18, 24, 36, 48, and 60 months post operation (±10% of interval, eg, 60 ± 6 months was defined as 60 months post operation).

### 2.8 Evaluation of autonomic reflexes

Autonomic function test (AFT) data were extracted from the SD, but such data were only available from patients who had been referred to the VADC. The AFTs informed about the integrity of autonomic reflex arcs by evaluating blood pressure and heart rate (HR) changes in response to orthostatic stress after a 70° head‐up tilt and Valsalva manoeuvre.^13^ Because the charts were de‐identified, only the blood pressure and HR data obtained during the head‐up tilt and Valsalva manoeuvre were available for reporting. The original files needed for more in‐depth analyses of autonomic function were not available.

For comparison, 30 normal controls with no autonomic dysfunction or bariatric surgery (negative controls) and 30 patients with severe autonomic neuropathy (positive controls) were included; these groups were not age or body mass index (BMI) matched because autonomic neuropathy is a neurodegenerative condition that generally affects the elderly. To determine whether any detected autonomic neuropathy was a result of vitamin deficiency, levels of vitamins B1, B6, and B12 were also analysed.

### 2.9 Follow‐up data

Patient retention was calculated at defined time points: 1, 2, 3, 4, and 5 years post operation. Patient retention for each patient at each time point was defined by whether recorded medical encounters existed in the VMBSD during the corresponding time interval between consecutive time points.

### 2.10 Statistical analyses

Baseline preoperative comorbidity and demographic data were summarized for the subset of patients who developed OI and for the rest of the study bariatric surgery population. Discrete data were summarized as percentages, and comparisons between the groups were made using the Chi‐square test. To examine the potential association between weight changes and the development of OI, we plotted the weight trends for all patients with bariatric surgery and for patients who developed OI against a rug plot of onset time for each patient identified as a case of OI. For this assessment, trends in weight for each group were expressed as excess body weight lost, in which excess weight was determined using ideal weights defined by Robinson's formula (Appendix S3). Finally, to quantify the development of new‐onset OI among patients who underwent bariatric surgery, the cumulative incidence of OI was calculated using a life table approach.^14^ For this calculation, the risk of developing OI was calculated for each year of follow‐up post bariatric surgery and by the end of 5 years post operation. This approach takes into account losses to follow‐up, and patients who were lost to follow‐up during a specific time interval (ie, a year) were assumed to have remained at risk and under observation for half that period.^14^ All analyses were performed using SAS 9.1 (SAS Institute Inc, Cary, NC) or R version 3.2.1 (The R Foundation for Statistical Computing).

## 3 RESULTS

### 3.1 Patients with bariatric surgery

Between 1997 and 2017, 4547 patients underwent bariatric surgery operations at VUMC. Of those patients, 973 underwent VSG (21.4%) and 3574 underwent RYGB (78.6%).

### 3.2 Identification of patients with OI

**Figure 1:**
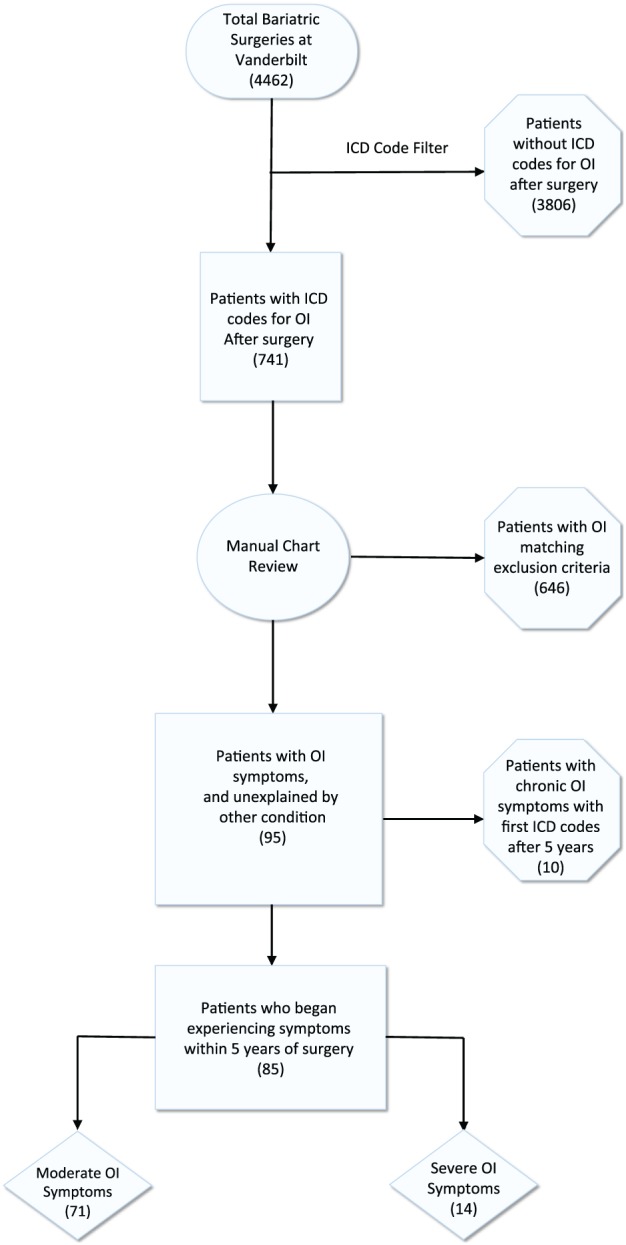
Retrospective orthostatic intolerance (OI) identification flowchart. Graphic representation of the workflow used for the identification of OI cases. The flowchart showed the steps as boxes in sequential order on the basis of the exclusion of non‐OI cases

Among the 4547 bariatric surgery patients, 741 (16.3%) patients were identified as potential cases of OI. After detailed medical records review and adjudication, 85 patients met our definition of postoperative new‐onset OI. Twelve (14%) of these patients had undergone VSG, and 73 (86%) had undergone RYGB. Of these 85 patients, 14 (16.5%) required vasopressors (midodrine and or fludrocortisone) for management and were considered severe cases because nonmedication measures to control symptoms (ie, fluid intake or salt) did not suffice to control the symptoms (Figure 1).

### 3.3 Identification of comorbidity and demographic data

Demographic and comorbidity data of the postoperative new‐onset OI patients and corresponding data from all other bariatric surgery patients are displayed in Table 1. There were no significant differences in demographics between the groups; however, the prevalence of preoperative neuropathy and coronary artery disease was significantly higher among patients with new‐onset OI. Demographic and comorbidity data from the OI patients (n = 85) were also compared between patients with OI designated as “moderate” or “severe” (Table 2). No significant differences were observed between these groups.

**Table 1:** Baseline demographic and clinical characteristics of bariatric surgery patients

Parameter	Post‐Bariatric OI (n = 85)	All Other Patients (n = 4547)	*P* Value (Chi‐Squared)
Age, mean (SD), y	46.3 (10.9)	45.3 (11.0)	.41
Weigh, mean (SD), years	136.0 (28.9)	140.0 (30.4)	.23
Male no. (%)	17 (20.0)	1004 (22.5)	.58
Female no. (%)	68 (80.0)	3458 (77.5)	.58
White no. (%)	72 (84.7)	3641 (81.6)	.47
Black no. (%)	13 (15.3)	651 (14.6)	.86
Hispanic no. (%)	‐‐‐	58 (1.3)	.29
RYGB no. (%)	73 (85.9)	3507 (78.6)	.10
VSG no. (%)	12 (14.1)	955 (21.4)	.10
Hypertension no. (%)	57 (67.1)	3284 (73.6)	.18
Sleep apnoea no. (%)	63 (74.1)	2909 (65.2)	.09
Neuropathy no. (%)	9 (10.6)	203 (4.55)	.01
CHF no. (%)	8 (9.4)	272 (6.1)	.22
ESRD no. (%)	1(1.2)	49 (1.1)	.95
Diabetes no. (%)	33 (38.8)	1879 (42.1)	.55
CAD no. (%)	12 (14.1)	357 (8.0)	.04
Hypoglycaemia no. (%)	1 (1.2)	45 (1.0)	.87

*Note*. Data were abstracted from the Vanderbilt Synthetic Derivative.

Abbreviations: CAD, coronary artery disease; CHF, congestive heart failure; ESRD, end‐stage renal disease; OI, orthostatic intolerance; RYGB, Roux‐en‐Y gastric bypass; SD, standard deviation; VSG, vertical sleeve gastrectomy.

**Table 2:** Baseline demographic and clinical characteristics of patients who underwent bariatric surgery by severity

Parameter	Severe OI N = 14	Moderate OI n = 71	*P* Value (Chi‐Squared)
Age, mean (SD), y	46.4 (12.5)	46.3 (10.9)	
Weigh, mean (SD), years	135.1 (23.1)	136.3 (30.4)	
Male no. (%)	4 (28.6)	4 (18.3)	.43
Female no. (%)	10 (71.4)	58 (81.7)	.69
White no. (%)	78.6	61 (85.9)	.49
Black no. (%)	21.4	10 (14.1)	.31
Hispanic no. (%)	‐‐‐	‐‐	‐‐‐
Hypertension no. (%)	9 (64.3)	48 (67.6)	.89
Sleep apnoea no. (%)	12 (85.7)	51 (71.8)	.58
Neuropathy no. (%)	1 (7.1)	13 (18.3)	.35
CHF no. (%)	2 (14.3)	9 (12.7)	.88
ESRD no. (%)	1 (7.1)	‐‐‐	‐‐‐
Diabetes no. (%)	6 (42.9)	6 (38.0)	.79
CAD no. (%)	2 (14.3)	2 (14.1)	.98
Hypoglycaemia no. (%)	‐‐	1 (1.41)	‐‐‐

*Note*. Data were abstracted from the Vanderbilt Synthetic Derivative.

Abbreviations: CAD, coronary artery disease; CHF, congestive heart failure; ESRD, end‐stage renal disease; OI, orthostatic intolerance; RYGB, Roux‐en‐Y gastric bypass; SD, standard deviation; VSG, vertical sleeve gastrectomy.

### 3.4 Body weight data

**Figure 2:**
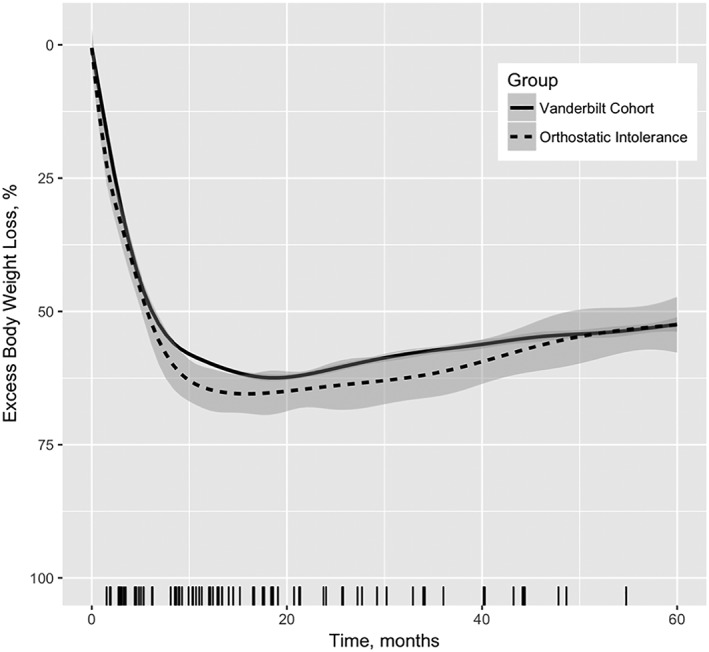
Mean excess body weight loss 5 years post operation of orthostatic intolerance (OI) patients vs all Vanderbilt bariatric surgery population. Shaded area indicates 95% confidence interval of the mean. Each tick on *X*‐axis indicates unique time of OI onset per patient

Percent excess body weight loss in patients with OI was compared against that of the complete bariatric cohort. Trend lines were modelled using a generalized polynomial regression of available percent excess weight loss data. No significant difference in percent excess body weight loss was detectable between the OI patients and the entire bariatric surgery cohort at Vanderbilt (Figure 2).

The weight loss trends against the onset of OI among patients was compared by juxtaposing the percent excess weight loss trendlines overtime against a rug plot of OI onset, where each tick represented a patient meeting our criteria for the first time in their records (Figure 2). This demonstrated OI symptom onset in a bimodal distribution; the first cluster occurred during initial months of greater weight loss, whereas the second trend occurred during stable‐weight months.

### 3.5 Cumulative incidence of OI

Among 4547 patients who underwent bariatric surgery, the team identified 85 cases (1.9%) of post‐bariatric OI in a 5‐year follow‐up period. Of note, the follow‐up rate at 5 years was only 17% (782 out of 4547); the life‐table calculation accounted for this loss of follow‐up during the 2‐ and 5‐year study period. The number of patients observed and lost to follow‐up and the number of patients with new‐onset OI during each annual time interval are shown in Table 3. The 2‐year cumulative incidence of post‐bariatric OI patients adjusted by loss of follow‐up was 2.2%, which is a conservative estimate. The estimated 5‐year cumulative incidence adjusted by loss of follow‐up was 4.2%.

**Table 3:** Life table

Years Since Surgery	No OI at Beginning of Year	First Reported Symptoms in Year	Loss of Follow‐up in Year	At Risk of Post‐Surgery OI During Year^a^	Proportion of Patients Beginning to Experience Symptoms during Year	Proportion Without OI During Year	Cumulative Proportion With no OI	Cumulative Proportion with OI
1	4547	45	1910	3592	0.0125	0.987	0.987	0.013
2	2592	20	1182	2001	0.0100	0.990	0.978	0.022
3	1390	10	409	1186	0.0084	0.992	0.969	0.031
4	971	7	182	880	0.0080	0.992	0.962	0.038
5	782	3		782	0.0038	0.996	0.958	0.042

*Note*. Data abstracted from Vanderbilt Synthetic Derivative and Vanderbilt Metabolic and Bariatric Surgery Quality, Efficacy, and Safety Database.

Abbreviation: OI, orthostatic intolerance.

Number of patients at risk for each time interval is calculated by subtracting half of the patients lost to follow‐up during a given interval from the number of patients without OI at the beginning of that interval.

### 3.6 Evaluation of autonomic function

**Figure 3:**
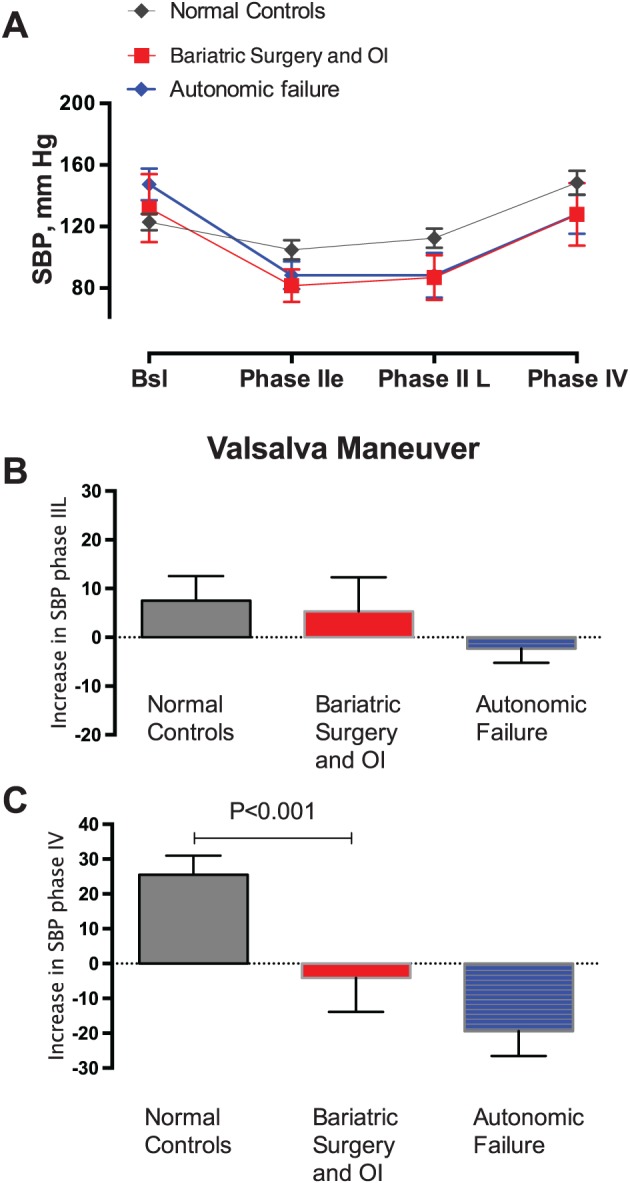
Bariatric surgery and orthostatic intolerance (OI) patients have decreased sympathetic nervous system (SNS) vasoconstrictor activity. The graphic represents continuous blood pressure and heart rate measurements during a Valsalva manoeuvre (VM). The pressor response in phase II late and phase IV represents sympathetic vasoconstrictor activity, which is key for the maintenance of orthostatic tolerance. We included a set of normal controls (N = 30, negative controls) and autonomic failure patients (N = 30, positive controls) for comparison. A, Systolic blood pressure during the four phases of VM in normal controls (N = 30, negative controls), bariatric surgery with OI (n = 10), and autonomic failure patients (n = 30, positive controls). B,C, Change in systolic blood pressure from baseline during phase II late and phase IV, respectively, of the VM. Patients with bariatric surgery and OI have impaired sympathetic vasoconstriction

AFT data were available for 21 (24.7%) of the 85 OI patients. Supine blood pressure was 122 ± 5.4/69 ± 2.9 mm Hg, HR 63 ± 2.2 bpm; blood pressure at 10 minutes after head‐up tilt at 70° were 126 ± 5.9/76 ± 3.0 mm Hg, HR 78 ± 2.8 bpm. Only five patients met the definition of orthostatic hypotension. No syncopal events were observed, eight of 21 (38%) had impaired sinus arrhythmia ratio, and 11 of 21 (52%) of these patients showed attenuated sympathetic vasoconstriction response as determined by the lack of increase in the pressor response in phase II late and phase IV of the Valsalva manoeuvre. These attenuated responses were quantified as changes in systolic blood pressure that closely resembled those of an external comparison group of patients with autonomic failure but differ significantly from the responses observed among another external comparison group with normal autonomic function, Figure 3.

Considering that autonomic neuropathy may be associated with vitamin deficiencies, the levels of vitamins B1, B6, and B12 were determined among patients with abnormal AFTs within the first 5 years post operation; vitamin B1 and B6 levels were within normal limits, whereas vitamin B12 level was persistently low in only one patient (Figure S1).

## 4 DISCUSSION

This is the first study to estimate the cumulative incidence of OI among patients who underwent bariatric surgery. We estimated that 4.2% of patients developed OI within 5 years of their operation. Furthermore, 16.5% of those OI cases had severe symptoms that warranted treatment with vasopressor agents.

Few studies have explored the long‐term postoperative consequences of bariatric surgery outside of weight loss. Most studies were largely focused on postoperative hypoglycemia.^15^, ^16^ A recent longitudinal study by Jakobsen et al,^10^ which followed a bariatric surgery patient cohort (n = 1888) for 6 years post operation, did not report OI as a significant adverse event. Instead, the study largely focused on medications prescribed and procedures done to examine adverse events related to surgical complications, re‐interventions, malnutrition, nausea, vomiting, and hypoglycaemia.

In recent years, multiple reports have documented occurrences of OI in patients after bariatric surgery^5^, ^6^, ^7^, ^17^; however, the prevalence and incidence of OI in this population were unknown. The ascertainment of OI is challenging because it is considered a diagnosis of exclusion. During our review, a number of patients were identified that presented with postsurgical presyncopal symptoms. These symptoms could be related to complications of the operation (eg, acute blood loss) or the use of vasoactive medications. Hence, we needed to perform a comprehensive medical chart reviews with a prespecified protocol to adjudicate the potential cases. Our review took into account the chronicity of symptoms, the comorbid conditions, and the medication use. This review was only possible with the use of the SD that allowed efficient access to and systematic review of de‐identified medical records.

The mechanism underlying post–bariatric surgery OI remains unknown. Our group^18^ and others^19^, ^20^ have shown that obesity results in an increased sympathetic (SNS) activity, decreased parasympathetic (PNS) activity, and altered baroreflex sensitivity. Previous studies have shown that bariatric surgery significantly reduces sympathetic activity, with VSG decreasing SNS as measured by direct sympathetic nerve recording at 6 and 12 months post operation.^21^ Similarly, RYGB also reduces SNS at 3 and 6 months post operation.^22^ Consistent with these observations, we observed patients with confirmed OI among those who underwent both VSG and RYGB. Although the mechanisms underlying these sympatholytic actions were not completely understood, these were thought to be associated with a decrease in visceral fat^23^ and leptin levels.^21^

Regardless of the mechanisms involved, the reduction in SNS activity induces a decline in blood pressure in postsurgical patients,^24^ which can have deleterious effects. For instance, after assuming an upright position, gravitational forces shift about 700 mL of blood volume to the lower part of the body, particularly the splanchnic circulation.^25^ To compensate for decreased venous return, the SNS is activated to induce vasoconstriction. These actions are fundamental for the maintenance of orthostatic tolerance. It is noteworthy that, among post‐bariatric patients with OI who had specialized autonomic testing, our group found evidence of impaired SNS activation as shown by the lack of increase in the pressor response during phase II late and phase IV of the Valsalva manoeuvre, Figure 3. This impaired autonomic response could be explained by the presence of autonomic neuropathy.

The prevalence of neuropathy as defined by the use of gabapentin and abnormal EMG was higher in the OI population, Table 1; even though the prevalence of neuropathy is low (10.6% in OI and 4.5% in all RYGB patients), it could be possible that these patients may have a subclinical autonomic neuropathy unmasked by the substantial weight loss after the operation.

New‐onset neuropathy associated with vitamin deficiency is less likely to explain OI considering that our cohort had normal vitamin levels during follow‐up. The prevalence of coronary artery disease and diabetes mellitus was also higher in the OI group; the significance of this finding is uncertain, and further studies are needed to investigate how this impacts the development of OI after bariatric surgery. Diabetes mellitus improves after bariatric surgery; therefore, worsening of diabetes‐induced neuropathy is not expected. In our cohort, the OI group had a tendency towards a higher prevalence of sleep apnoea. Sleep apnoea is associated with increased SNS activity and attenuated baroreflex regulation; it could be possible that these patients are at higher risk of developing OI because autonomic compensatory mechanisms to standing are attenuated prior to surgery. This hypothesis, however, need to be further studied.

The decrease in SNS could result from the substantial weight loss that occurred after the operation. Previous studies have shown that weight loss from hypocaloric diet results in reduced sympathetic activity as measured by muscle sympathetic nerve activity, which contributes to decreased orthostatic tolerance.^26^ Our data in 85 post–bariatric surgery subjects (Figure 3) depict time‐dependent incidence of OI. The data show that many OI patients had relatively stable weight at the time of symptom development. Finally, bariatric surgery may induce a persistent reduction in SNS activity that is not totally dependent on weight loss, contributing to the development of OI in susceptible subjects.

Given the retrospective and observational nature of the study, a causative relationship between bariatric surgery and OI cannot be established. Moreover, the retrospective format means relying on patient records and charts that may have incomplete or missing data on the clinical condition of the patient. The study was conducted at a major surgical centre in the Southeast United States, with many patients coming from other regions to the centre for their operation. Many patients continued to receive health care elsewhere, as demonstrated with important losses to follow‐up during the study period. Using the life table, we attempted to account for losses to follow‐up. As with other survival analysis techniques, this approach assumes that the risk of OI among those who left the cohort is similar to the risk in those who remained under observation. As patients who were lost to follow‐up were by definition no longer observable, there was no practical way to fully test this assumption. Nevertheless, our study examined a large cohort of patients who underwent bariatric surgery at a major surgical centre, and we identified patients who developed OI through review of medical records and the use of strict operational case definition. We provide estimates of incidence OI post operation while taking into account losses to follow‐up. Additional studies in other settings would be useful to complement our findings.

In conclusion, every year, over 200 000 individuals undergo bariatric surgery for the treatment of morbid obesity in the United States. OI characterized by chronic presyncopal symptoms with evidence of impaired sympathetic vasoconstriction activity is frequent in the bariatric population, affecting 4.2% of patients within the first 5 years postoperatively.

## FUNDING

C.A.S. was supported by the 2018 American Heart Association Innovative Project Award 18IPA34110172 and by Food and Drug Administration grant R01 FD04778‐02. R.A.T. was supported by National Institute of Health (NIH) grant R01 DK100431. V.L.A. was supported by F32 DK103474. C.G.G. was supported by the NIH National Institute on Aging grant R01 AG043471. The datasets used for the analyses described were obtained from Vanderbilt University Medical Center's Synthetic Derivative, which is supported by institutional funding and by the Vanderbilt CTSA grant ULTR000445 from NCATS/NIH.

## CONFLICT OF INTEREST STATEMENT

The authors have nothing to disclose.

## Supporting information

Supporting info item

Supplemental Figure 1. Vitamins B1, B6 and B12 trends in OI patients with impaired sympathetic vasoconstriction. Patient's values are plotted individually, and normal vitamin levels are denoted with a dashed line.
Appendix 1: ICD9/10 Codes for Symptoms of Orthostatic Intolerance
Appendix 2: ICD9/10 Codes for Comorbidities
Appendix 3: Robinson's Ideal Body Weight Equation
