# Supplementary material for: The incidence of orthostatic intolerance after bariatric surgery
Source: Obes Sci Pract. 2019 Dec 6;6(1):76–83. doi: 10.1002/osp4.383 (PMC7042102; doi:10.1002/osp4.383)
Supplement: Supplementary file 2 — Supplemental Figure 1. Vitamins B1, B6 and B12 trends in OI patients with impaired sympathetic vasoconstriction. Patient's values are plotted individually, and normal vitamin levels are denoted with a dashed line. Appendix 1: ICD9/10 Codes for Symptoms of Orthostatic Intolerance Appendix 2: ICD9/10 Codes for Comorbidities Appendix 3: Robinson's Ideal Body Weight Equation [file OSP4-6-76-s002.docx]

**The Incidence of Orthostatic Intolerance After Bariatric Surgery**

James B. Zhang, BA^2^; Robyn A. Tamboli, PhD^2^; Vance L. Albaugh, MD, PhD^2^; Carlos G. Grijalva, MD, MPH^3, 4^, David B. Williams, MD^2^, Donna M. Kilkelly, PA^2^; Cyndya A. Shibao, MD, MSCI^1^

^1^ Department of Medicine, Vanderbilt University Medical Center, Nashville, TN 37232

^2^ Department of Surgery, Vanderbilt University Medical Center, Nashville, TN 37232

^3^ Department of Health Policy, Vanderbilt University Medical Center, Nashville, TN 37232

^4^ Mid-South Geriatric Research Education, and Clinical Center, VA Tennessee Valley Health Care System, Nashville, TN 37212

**Supplemental Material**

*Number of tables: 1*

*Number of figures: 1*

*Appendix 1*

*Appendix 2*

**
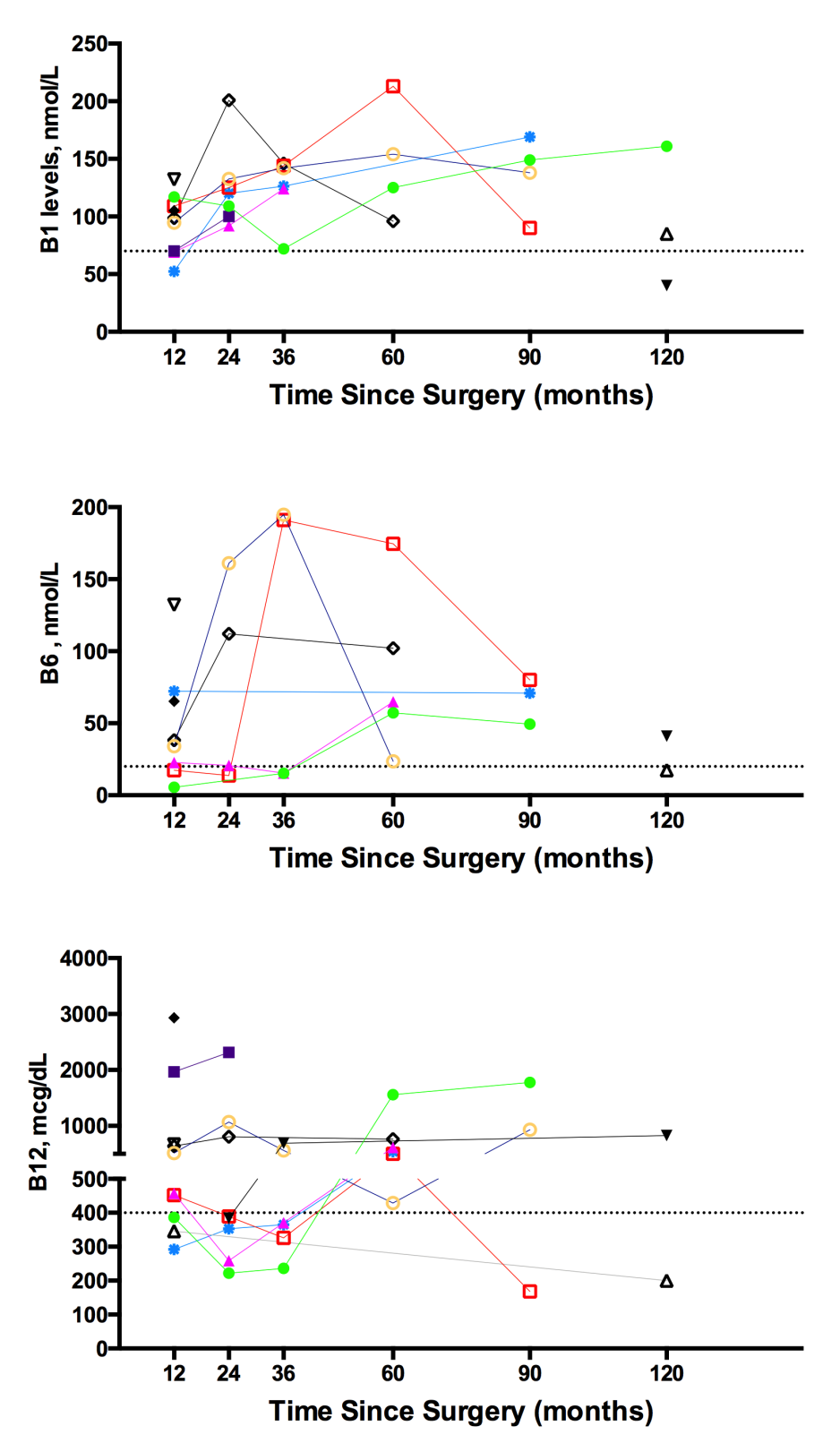
**

**Supplemental Figure 1.** Vitamins B1, B6 and B12 trends in OI patients with impaired sympathetic vasoconstriction. Patient’s values are plotted individually, and normal vitamin levels are denoted with a dashed line.

**Appendix 1: ICD9/10 Codes for Symptoms of Orthostatic Intolerance**

- Dizziness and Giddiness
  - ICD9: 780.4 Dizziness and Giddiness
  - ICD10: R42 Dizziness and Giddiness
- Syncope
  - ICD9: 780.2 Syncope and Collapse
  - ICD10: R55 Syncope and Collapse
- Orthostatic Hypotension
  - ICD9: 458.0 Orthostatic Hypotension, 458.9 Hypotension, Unspecified
  - ICD10: I95.1 Orthostatic Hypotension, I95.9 Hypotension, Unspecified
- Tachycardia
  - ICD9: 427 Cardiac Dysrhythmias, 427.2 Paroxysmal Tachycardia, Unspecified, 785.0 Tachycardia, Unspecified
  - ICD10: I47.1 Supraventricular Tachycardia, I47.9 Paroxysmal Tachycardia, R00.0 Tachycardia, Unspecified
- Bradycardia
  - ICD9: 427.89 Other Specified Cardiac Dysrhythmias, 427 Cardiac Dysrhythmia
  - ICD10: R00.1 Bradycardia, Unspecified

**Appendix 2: ICD9/10 Codes for Comorbidities**

Hypertension

ICD9: 401, 401.0, 401.1, 401.9

ICD10: I10

Sleep Apnea

ICD9: 327.2, 327.20, 327.21, 327.23, 327.27, 327.29, 780.57, 786.03

ICD10: G47.30, G47.31, G47.33, G47.37, G47.39, R06.81

Neuropathy

ICD9: 337.0, 337.00, 337.09, 337.1, 356.4, 356.8, 357.2

ICD10: G60.3, G62.9, G90.09

Congestive Heart Failure

ICD9: 402.01, 402.11, 402.91, 404.01, 404.03, 404.11, 404.13, 404.91, 404.93, 428, 428.0, 428,1, 428.20, 428.22, 428.30, 428.32, 428.9

ICD10: I11.0, I13.0, I13.2, I50.20, I50.21, I50.22, I50.23, I50.30, I50.31, I50.32, I50.33, I50.40, I50.41, I50.42, I50.43, I50.9

End Stage Renal Disease

ICD9: 403.01, 403.11, 403.91, 403.91, 404.02, 404.03, 404.12, 404.13, 404.92, 404.93, 585.6

ICD10: I12.0, I13.11, I13.2, N18.6

Type II Diabetes

ICD9: 250, 250.0, 250.00, 250.02, 250.1, 250.10, 250.12, 250.2, 250.20, 250.22, 250.3, 250.30, 250.32, 250.4, 250.40, 250.42, 250.5, 250.50, 250.52, 250.6, 250.60, 250.62, 250.7, 250.70, 250.72, 250.8, 250.80, 250.82, 250.9, 250.90, 250.92

ICD10: E11.00, E11.01, E11.21, E11.22, E11.29, E11.311, E11.319, E11.3211, E11.3212, E11.3213, E11.3219, E11.3291, E11.3292, E11.3293, E11.3299, E11.3311, E11.3312, E11.3313, E11.3319, E11.3391, E11.3392, E11.3393, E11.3399, E11.3411, E11.3412, E11.3413, E11.3419, E11.3491, E11.3492, E11.3493, E11.3499, E11.3511, E11.3512, E11.3513, E11.3519, E11.3521, E11.3522, E11.3523, E11.3529, E11.3531, E11.3532, E11.3533, E11.3539, E11.3541, E11.3542, E11.3543, E11.3549, E11.3551, E11.3552, E11.3553, E11.3559, E11.3591, E11.3592, E11.3593, E11.3599, E11.36, E11.37X1, E11.37X2, E11.37X3, E11.37X9, E11.39, E11.40, E11.41, E11.42, E11.43, E11.44, E11.49, E11.51, E11.52, E11.59, E11.610, E11.619, E11.620, E11.621, E11.622, E11.628, E11.630, E11.638, E11.641, E11.649, E11.65, E11.69, E11.8, E11.9

Coronary Artery Disease

ICD9: 414.0, 414.00, 414.01

ICD10: I25.10, I25.110, I25.111, I25.118, I25.119

Hypoglycemia

ICD9: 251.1, 251.2

ICD10: E16.1, E16.2

**Appendix 3: Robinson’s Ideal Body Weight Equation**

Ideal Body Weight (men) = 52kg +1.9 (kg/inch over 5 feet)

Ideal Body Weight (women) = 49 kg + 1.7 (kg/inch over 5 feet)
