# Supplementary material for: The incidence of orthostatic intolerance after bariatric surgery
Source: Obes Sci Pract. 2019 Dec 6;6(1):76–83. doi: 10.1002/osp4.383 (PMC7042102; doi:10.1002/osp4.383)
Supplement: Supplementary file 1 — Supporting info item [file OSP4-6-76-s001.pdf]

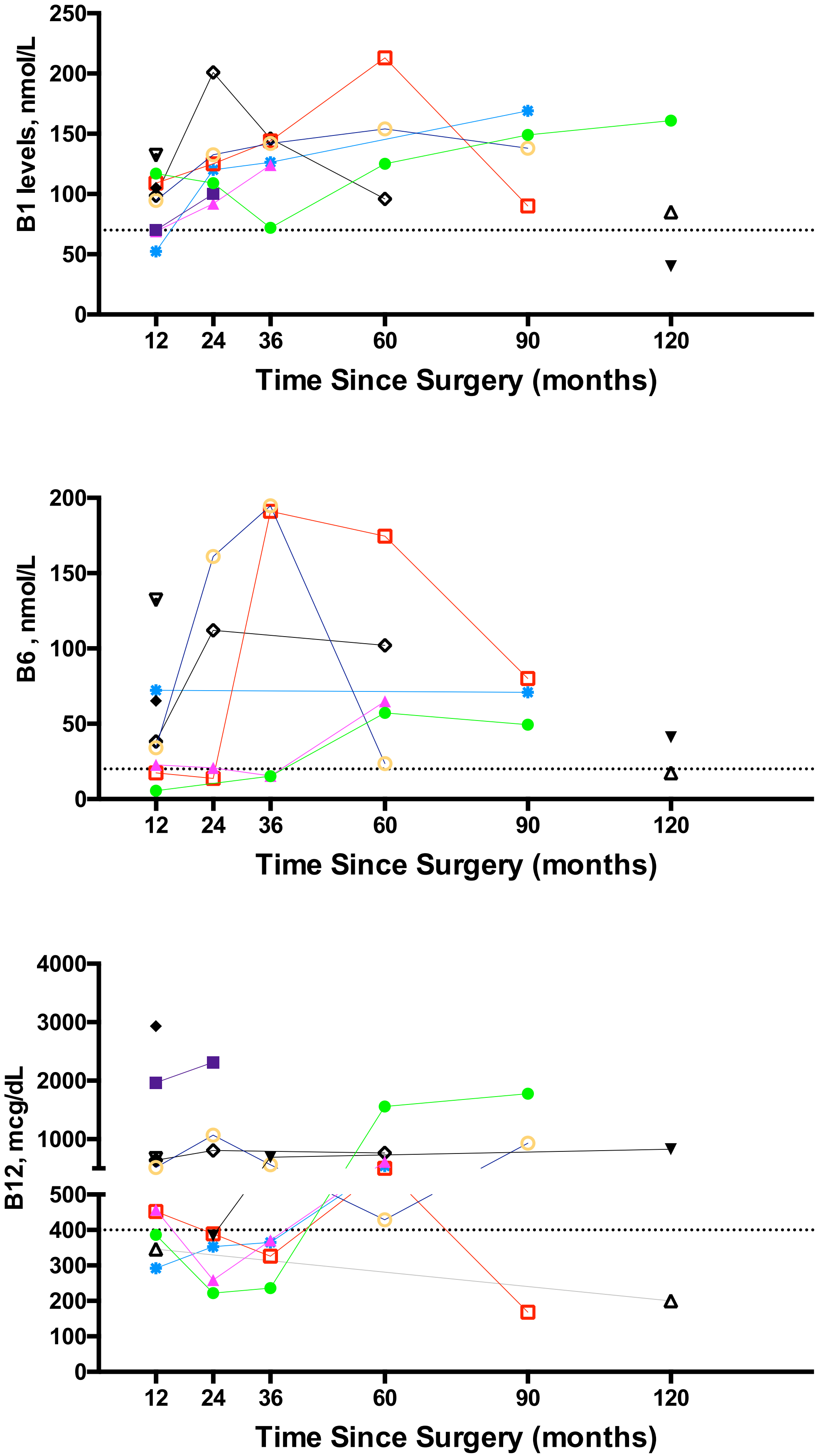

B1 levels, nmol/L
250
200
150
100
50
0
12
24
36
60
90
120
Time Since Surgery (months)
B6 , nmol/L
200
150
100
50
0
12
24
36
60
90
120
Time Since Surgery (months)
B12, mcg/dL
4000
3000
2000
1000
500
400
300
200
100
0
12
24
36
60
90
120
Time Since Surgery (months)
